# Transplant centers that assess frailty as part of clinical practice have better outcomes

**DOI:** 10.1186/s12877-022-02777-2

**Published:** 2022-01-27

**Authors:** Xiaomeng Chen, Yi Liu, Valerie Thompson, Nadia M. Chu, Elizabeth A. King, Jeremy D. Walston, Jon A. Kobashigawa, Darshana M. Dadhania, Dorry L. Segev, Mara A. McAdams-DeMarco

**Affiliations:** 1 Department of Surgery, Johns Hopkins University School of Medicine, 2000 E Monument Street, Baltimore, MD 21205 USA; 2 Department of Epidemiology, Johns Hopkins Bloomberg School of Public Health, Baltimore, MD USA; 3 Department of Medicine, Johns Hopkins School of Medicine, Baltimore, MD USA; 4 Comprehensive Transplant Center, Cedars-Sinai Medical Center, Los Angeles, CA USA; 5 Division of Nephrology and Hypertension, Weill Cornell Medicine, New York, NY USA

**Keywords:** Frailty, Kidney Transplant, Mortality, Graft Loss, Clinical Practice

## Abstract

**Background:**

Frailty predicts adverse post-kidney transplant (KT) outcomes, yet the impact of frailty assessment on center-level outcomes remains unclear. We sought to test whether transplant centers assessing frailty as part of clinical practice have better pre- and post-KT outcomes in all adult patients (≥18 years) and older patients (≥65 years).

**Methods:**

In a survey of US transplant centers (11/2017–4/2018), 132 (response rate = 65.3%) centers reported their frailty assessment practices (frequency and specific tool) at KT evaluation and admission. Assessment frequency was categorized as never, sometime, and always; type of assessment tool was categorized as none, validated (for post-KT risk prediction), and any other tool. Center characteristics and clinical outcomes for adult patients during 2017–2019 were gleaned from the transplant national registry (Scientific Registry of Transplant Recipients). Poisson regression was used to estimate incidence rate ratios (IRRs) of waitlist outcomes (waitlist mortality, transplantation) in candidates and IRRs of post-KT outcomes (all-cause mortality, death-censored graft loss) in recipients by frailty assessment frequency. We also estimated IRRs of waitlist outcomes by type of assessment tool at evaluation. All models were adjusted for case mix and center characteristics.

**Results:**

Assessing frailty at evaluation was associated with lower waitlist mortality rate (always IRR = 0.91,95%CI:0.84–0.99; sometimes = 0.89,95%CI:0.83–0.96) and KT rate (always = 0.94,95%CI:0.91–0.97; sometimes = 0.88,95%CI:0.85–0.90); the associations with waitlist mortality rate (always = 0.86,95%CI:0.74–0.99; sometimes = 0.83,95%CI:0.73–0.94) and KT rate (always = 0.82,95%CI:0.77–0.88; sometimes = 0.92,95%CI:0.87–0.98) were stronger in older patients. Furthermore, using validated (IRR = 0.90,95%CI:0.88–0.92) or any other tool (IRR = 0.90,95%CI:0.87–0.93) at evaluation was associated lower KT rate, while only using a validated tool was associated with lower waitlist mortality rate (IRR = 0.89,95%CI:0.83–0.96), especially in older patients (IRR = 0.82,95%CI:0.72–0.93). At admission for KT, always assessing frailty was associated with a lower graft loss rate (IRR = 0.71,95%CI:0.54–0.92) but not with mortality (IRR = 0.93,95%CI:0.76–1.13).

**Conclusions:**

Assessing frailty at evaluation is associated with lower KT rate, while only using a validated frailty assessment tool is associated with better survival, particularly in older candidates. Centers always assessing frailty at admission are likely to have better graft survival rates. Transplant centers may utilize validated frailty assessment tools to secure KT access for appropriate candidates and to better allocate health care resources for patients identified as frail, particularly for older patients.

**Supplementary Information:**

The online version contains supplementary material available at 10.1186/s12877-022-02777-2.

## Background

Frailty, originally characterized in community-dwelling older adults, is a clinical phenotype of decreased physiologic reserve and resistance to stressors [1]. Accumulating evidence has suggested that frailty may be a more meaningful predictor of mortality and hospitalizations than age among dialysis patients [2]. While a number of tools have been developed for frailty assessment over the past decades, the most commonly studied tool among end-stage kidney disease (ESKD) patients is the Physical Frailty Phenotype (PFP) developed by Fried et al. [3]. Frailty measured by the PFP is common in kidney transplant (KT) populations; 16% of KT candidates and 14% of KT recipients are frail in the United States [4]. Recently, frailty has gained the attention of renal healthcare providers for its ability to predict adverse outcomes among adult individuals with ESKD and KT patients [2, 5–10]. Among KT candidates, frailty is associated with lower chance of listing, higher waitlist mortality, and reduced access to KT [11–14]; among KT recipients, frailty is associated with surgical complications, delayed graft function, postoperative delirium, early hospital readmission, immunosuppression intolerance, and mortality [15–21]. Other surrogates of frailty that focus on physical function, such as the Short Physical Performance Battery (SPPB), functional status, Kidney Disease Quality of Life Short Form Physical Component Subscale (SF-12 PCS), gait speed, timed up and go, have also been found to predict adverse post-KT outcomes among ESKD and KT patients [22–28]. Another major frailty framework is the deficit accumulation model (Frailty Index) developed by Mitnitski et al., who viewed frailty as a state of accelerated deficit accumulation [29]. The frailty index has shown similar predictive value to PFP for clinical outcomes [30] but has yet to be applied to KT populations.

The American Society of Transplantation (AST) Kidney/Pancreas Community of Practice Workgroup conducted a survey of US KT programs in 2017–2018 to assess the landscape of frailty assessments at transplant centers in the United States. According to the survey, frailty is recognized as a clinically relevant construct in candidacy evaluation due to its potential in risk stratification for pre- and post-KT outcomes [6]. Particularly, measuring frailty is useful for identifying which older candidates are robust despite their age. In practice, frailty assessments are used in approximately 70% of transplant centers during candidacy evaluation and in approximately 30% of centers at KT admission [6].

Despite the use of frailty in the clinical practice of some transplant centers, it is unclear whether assessing frailty at evaluation and/or admission impacts pre- and post-KT outcomes of transplant centers. We hypothesized that transplant centers may take the advantage of frailty assessment results to better allocate health care resources for patients identified as frail, to prioritize robust older candidates, and to identify candidates whose physiologic reserve may improve after KT, which may in turn contribute to better patient outcomes at these centers. In this study, we sought to test whether transplant centers that measure frailty as part of clinical practice have better pre- and post-KT clinical outcomes and whether the associations differ among older patients (≥65 years).

## Methods

### Study Design

During 11/2017–4/2018, the AST Kidney Pancreas Community of Practice Workgroup administered a survey about practices related to frailty among all transplant centers that performed adult KT in the United States in 2017. The adult transplant centers were identified from the Scientific Registry of Transplant Recipients (SRTR) external release. The SRTR data system includes data on all donors, waitlisted candidates, and transplant recipients in the United States submitted by members of the Organ Procurement and Transplantation Network (OPTN). The Health Resources and Services Administration, United States Department of Health and Human Services provides oversight to the activities of the OPTN and SRTR contractors. The survey was exempt by the Johns Hopkins School of Medicine Institutional Review Board, and this research is in adherence with the Declaration of Helsinki and the Declaration of Istanbul. Further details about this survey were described elsewhere [6].

Among the 202 adult KT centers, a total of 132 centers (response rate = 65.3%) reported their frequencies of frailty assessment administration at evaluation and at KT. We gleaned center characteristics and clinical outcomes of the responding centers on waitlisted candidates and transplant recipients in the United States during 2017–2019. Center-level mean characteristics of waitlist candidates included adult KT listing volume per year (i.e., number of unique adult patients waitlisted in the study period), mean age of candidates at the time of KT listing, percentage of candidates who were older (aged ≥65 years), percentage of female candidates, percentage of Hispanic candidates, percentage of Black candidates, percentage of candidates who had a highest education level of high school or less, percentage of candidates currently working for income, percentage of candidates with diabetes, and percentage of candidates on dialysis. Center-level mean characteristics of recipients included adult KT volume per year (i.e., number of unique adult KT recipients in the study period), mean age of recipients at the time of KT, percentage of recipients who were older, percentage of female recipients, percentage of Hispanic recipients, percentage of Black recipients, percentage of recipients who had a highest education level of high school or less, percentage of recipients currently working for income, percentage of recipients with diabetes, percentage of recipients on dialysis, and percentage of recipients who received a living donor KT.

To assess the center performance at the time of the survey, we linked the dataset with the biannual SRTR Program-Specific Report (PSR) released in 10/2018 to obtain center-specific observed to expected (O/E) ratio for each adverse outcome rate: waitlist mortality rate and transplantation rate for waitlist candidates; 1-year all-cause mortality race and 1-year graft loss rate for recipients. The reference population of the waitlist estimates was all KT patients on the waiting list at any time during 12/31/2015–12/30/2017. The reference population of the post-transplant estimates was all KT patients undergoing transplant during the same 2-year period. We also examined whether the responding transplant centers had a geriatrics programs gleaned from the official website of each center.

### Frailty Assessment Practice

The frequency of frailty assessment at candidacy evaluation and at KT admission were assessed by two questions: “Do you currently perform a standardized frailty assessment as part of evaluation for kidney transplant candidacy in your practice?” and “Do you currently perform a standardized frailty assessment for kidney transplant recipients at the time of transplantation in your practice?” The possible responses to either question included “always,” “sometimes,” and “never.” If the participants answered “always” or “sometimes” to either question, they were further asked “What tool for the assessment of frailty do you currently use routinely?” and were able to select all that applied from a list of common frailty assessment tools. The full list of the standardized tools and responses to this question were recently reported elsewhere [6]. We categorized frailty assessment tool as: none (i.e., never assessing frailty), validated tool, and any other tool. Based on the literature, we identified 5 validated frailty assessment tools for post-KT risk prediction, including PFP, SPPB, functional status (i.e., SF-12 PCS), timed walk (i.e., 6-min walk test), and timed up and go.

### Waitlist Outcomes

The center-level waitlist outcomes for candidates included waitlist mortality (i.e., death while awaiting a KT) rate and transplantation rate. Waitlisted candidates were followed from listing to removal from waitlist (due to death or KT) or administrative censoring at 8/2020. Total numbers of deaths and KTs were counted for each center. For the follow-up period of each candidate, person-years were calculated as the number of days that the candidate was on the waitlist and converted to a fraction of a year. Total person-years for each transplant center were calculated from the sum of person-years of each candidate waitlisted in 2017–2019 at the center. Waitlist mortality rate per 100 person-years on the waitlist was calculated by dividing the number of removals due to death at each center by the total number of person-years on the waitlist at the center, multiplied by 100. Transplantation rate per 100 person-years on the waitlist was calculated by dividing the number of removals due to KT at each center by the total number of person-years on the waitlist at the center, multiplied by 100.

### Post-KT Outcomes

The post-KT outcomes of interest included patient survival represented by all-cause mortality rate and graft survival represented by death-censored graft loss rate. KT recipients were followed for death and graft loss until administrative censoring at 8/2020. Total numbers of all-cause deaths and death-censored graft losses were counted for each center. Person-years were calculated as the number of days that the recipients spent until the outcome event (all-cause death or death-censored graft loss) and converted to a fraction of a year. Total person-years of each outcome event for each transplant center were calculated from the sum of person-years of each recipient in 2017–2019 at the center. All-cause mortality rate and death-censored graft loss rate per 100 person-years were calculated, respectively, by dividing the number of events at each center by the total number of person-years at the center, multiplied by 100.

### Primary Analysis

For candidates who had multiple listing records during 2017–2019 at each center, only the first listing record of each candidate was included for analysis; similarly, only the first transplant record of each recipient over the same period at each center was included. We used patient-level data from SRTR to estimate center-level mean characteristics. We generated means with standard deviations (SDs) for center-mean characteristics, medians with interquartile ranges (IQRs) for non-normally distributed continuous variables, and percentages for categorical variables by frailty assessment frequency. Differences in the distributions were tested using analysis of variance (ANOVA) for normally distributed variables, Kruskal-Wallis test for non-normally distributed variables, and Fisher’s exact test for categorical variables.

Unadjusted waitlist mortality rate and transplantation rate were calculated by frequency of frailty assessment at evaluation; unadjusted rates of all-cause mortality and death-censored graft loss were calculated by frequency of frailty assessment at KT. Crude and adjusted incidence rate ratios (cIRRs and aIRRs) of each event (waitlist mortality, transplantation, all-cause mortality, and death-censored graft loss) by frequency of frailty assessment were estimated using three Poisson regression models: crude models generated cIRRs; demographic and health factor models adjusted for center-mean demographic (percentage of older, percentage of female, percentage of Black, percentage of Hispanic) and health characteristics (percentage of patients with diabetes, percentage of undergoing dialysis, and percentage of recipients who received a living donor KT); demographic, health, and social factor models further adjusted for center-mean socio-economic characteristics (percentage of having high school or less education, and percentage of working for income). As a secondary analysis, we further examined the impact of the type of frailty assessment tool by estimating cIRRs and aIRRs of waitlist outcomes (waitlist mortality, transplantation) by frailty assessment tool at evaluation.

All analyses were performed using Stata version 15 (StataCorp, College Station, TX). Two-sided *p*-values <0.05 were considered statistically significant.

### Subgroup Analysis in Older Patients

We performed a subgroup analysis to evaluate the associations of frailty assessment practice with pre- and post-KT outcomes in older patients, specifically estimating 1) IRRs of waitlist mortality and transplantation by frailty assessment frequency and assessment tool at evaluation in older candidates, and 2) IRRs of all-cause mortality and death-censored graft loss by frailty assessment frequency at KT in older recipients.

## Results

### Center Characteristics of Candidates at Evaluation

During 2017–2019, the 132 responding centers listed 75.1% of the total adult KT candidates. These centers had a mean volume of adult KT listing of 206.0 (SD = 148.7) candidates per year during 2017–2019 and the center-mean age at listing was 52.9 years (SD = 2.0). The candidates listed at these centers were on average 21.2% older adults, 37.9% female, 16.5% Hispanic, 27.7% Black; 43.5% having high school or less education, and 37.5% working for income; 43.9% of candidates had diabetes and 70.1% were on dialysis at the time of listing. Center characteristics did not differ by frequency of frailty assessment at evaluation (all *p* > 0.05) (Table 1). There was no difference in median O/E ratios of waitlist mortality (never = 1.05, sometimes = 0.93, always = 0.98, *p* = 0.35) or transplantation (never = 1.06, sometimes = 0.92, always = 0.89, *p* = 0.22) by frailty assessment frequency (Table 1 and Supplementary Fig. S1). The proportion of centers having a geriatrics program was also similar by frailty assessment frequency (never = 66.7%, sometimes = 57.1%, always = 72.4%, *p* = 0.36) (Table 1).

**Table 1:** Characteristics of kidney transplant centers by frequency of frailty assessment at kidney transplant evaluation (*N* = 132)

Center characteristics	Frequency of frailty assessment at evaluation			
	Never (***N*** = 54)	Sometimes (***N*** = 49)	Always (***N*** = 29)	***P***-value
Adult KT listing volume per year	211.1 (140.9)	205.0 (173.4)	198.4 (119.3)	0.93
Age (years)	53.3 (2.0)	52.6 (1.5)	52.8 (2.4)	0.27
% Older (≥65 years)	22.1 (6.0)	20.2 (5.2)	21.3 (6.4)	0.26
% Female	37.6 (7.2)	38.9 (3.5)	36.7 (9.2)	0.34
% Hispanic	16.2 (16.6)	17.7 (20.5)	14.7 (18.6)	0.78
% Black	32.1 (19.0)	23.2 (16.6)	26.9 (21.1)	0.06
% High school or less	44.3 (10.8)	44.2 (9.9)	40.9 (9.0)	0.29
% Working for income	35.3 (9.4)	39.3 (7.6)	38.7 (9.0)	0.05
% Diabetes	45.3 (6.0)	43.0 (7.8)	42.9 (7.1)	0.19
% Undergoing dialysis	71.6 (11.0)	68.1 (11.4)	70.7 (9.2)	0.26
O/E ratio of waitlist mortality, median (IQR)	1.05 (0.84, 1.13)	0.93 (0.82, 1.10)	0.98 (0.85, 1.13)	0.35
O/E ratio of transplantation, median (IQR)	1.06 (0.77, 1.49)	0.92 (0.75, 1.24)	0.89 (0.68, 1.31)	0.22
Geriatrics program, n (%)	36 (66.7%)	28 (57.1%)	21 (72.4%)	0.36
Use of a validated frailty assessment tool, n (%)	–	33 (67.3%)	18 (62.1%)	0.64

Center characteristics were gleaned from SRTR data on adult KT candidates in 2017–2019. The table shows the mean center characteristics, including characteristics that are percentages. Values are presented as mean (SD) across transplant centers unless otherwise indicated. *Abbreviations*: *KT* Kidney transplantation, *SD* Standard deviation, *IQR* Interquartile range, *O/E ratio* Observed to expected ratio

### Frailty Assessment at Evaluation and Waitlist Outcomes

**Fig. 1:**
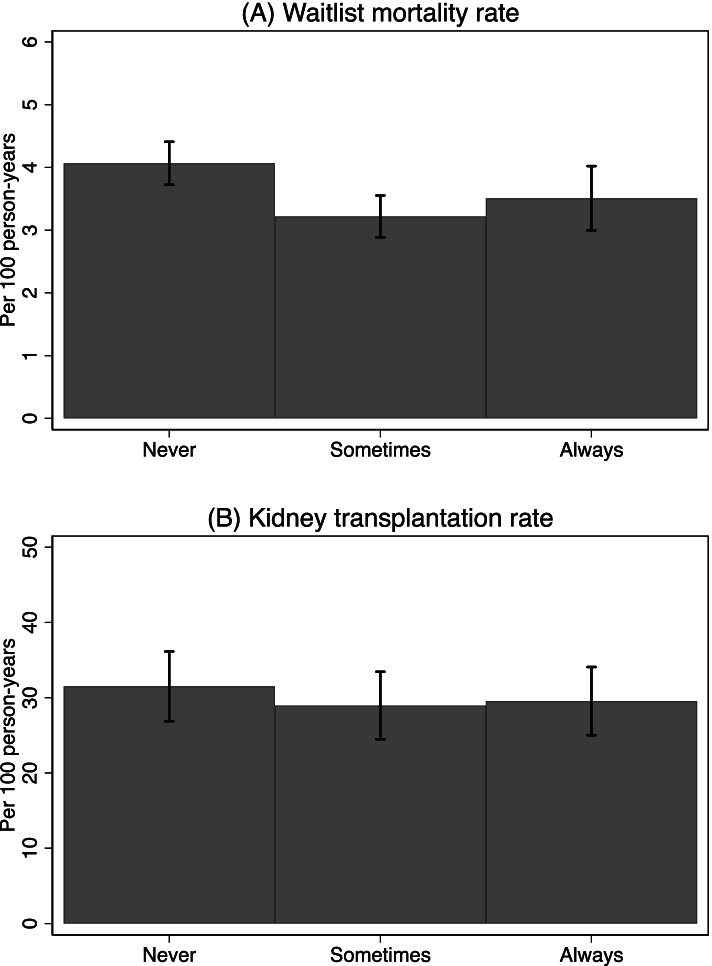
Unadjusted center-specific incidence rates among kidney transplant candidates by frequency of frailty assessment (*N* = 132)**.** Patient survival data was gleaned from SRTR data on adult KT candidates in 2017–2019. Error bars represent the corresponding 95% confidence intervals

Of the 132 KT centers, 22.0% (*n* = 29) always, 37.1% (*n* = 49) sometimes, and 40.9% (*n* = 54) never assessed frailty at KT candidacy evaluation. Among centers assessing frailty at evaluation, 65.4% (*n* = 51) used a validated frailty assessment tool. The average of center median wait time for a KT was 1.3 years (SD = 0.3); there was no difference in wait time by frequency of frailty assessment (*p* = 0.38). The centers had a mean waitlist mortality rate of 3.6 deaths per 100 person-years (SD = 1.3), with 4.1 deaths (SD = 1.3), 3.2 deaths (SD = 1.2), and 3.5 deaths (SD = 1.3) per 100 person-years for centers never, sometimes, and always assessing frailty at evaluation, respectively (*p* = 0.003) (Fig. 1A). The center-mean transplantation rate was 30.1 KTs per 100 person-years (SD = 15.4), and there was no difference in transplantation rates by frequency of frailty assessment (*p* = 0.69) (Fig. 1B).

After the adjustment for demographic, health, and social factors, centers always assessing frailty had a 9% lower incidence rate of waitlist mortality (aIRR = 0.91, 95% confidence interval [CI]: 0.84–0.99), and centers sometimes assessing frailty had a 11% lower incidence rate of waitlist mortality (aIRR = 0.89, 95% CI: 0.83–0.96). Furthermore, centers always assessing frailty had a 6% lower incidence rate of KT (aIRR = 0.94, 95% CI: 0.91–0.97), and centers sometimes assessing frailty had a 12% lower incidence rate of KT (aIRR = 0.88, 95% CI: 0.85–0.90) (Table 2).

**Table 2:** Frailty assessment at kidney transplant evaluation and center-specific waitlist mortality and transplantation rates (*N* = 132)

	Crude model	Demographic + health factor model	Demographic + health + social factor model
	cIRR (95% CI)	aIRR (95% CI)	aIRR (95% CI)
**Waitlist mortality rate**			
Never	reference	reference	reference
Sometimes	**0.84 (0.78, 0.90)**	**0.89 (0.82, 0.95)**	**0.89 (0.83, 0.96)**
Always	**0.86 (0.79, 0.93)**	**0.90 (0.83, 0.98)**	**0.91 (0.84, 0.99)**
**Transplantation rate**			
Never	reference	reference	reference
Sometimes	**0.89 (0.87, 0.92)**	**0.87 (0.85, 0.90)**	**0.88 (0.85, 0.90)**
Always	**1.07 (1.04, 1.10)**	**0.94 (0.91, 0.97)**	**0.94 (0.91, 0.97)**

Crude and adjusted incidence rate ratios (cIRR and aIRR) with 95% confidence intervals (CI) are presented from Poisson regression models. Demographic + health factor models adjusted for center-mean demographic (% older, % female, % Black, % Hispanic) and health factors (% with diabetes, % undergoing dialysis); demographic + health + social factor models additionally adjusted for center-mean social factors (% low education, % working for income). Associations that are statistically significant at *p* < 0.05 are bolded

Furthermore, independent of demographic, health, and social factors, using a validated frailty assessment tool at evaluation was associated with lower waitlist mortality (IRR = 0.89, 95% CI: 0.83–0.96) and lower KT rate (IRR = 0.90, 95% CI: 0.88–0.92), while using any other assessment tool was associated with lower KT rate (IRR = 0.90, 95% CI: 0.87–0.93) but not with waitlist mortality (IRR = 0.92, 95% CI: 0.84–1.00) (Table 3).

**Table 3:** Type of frailty assessment tool at kidney transplant evaluation and center-specific waitlist mortality and transplantation rates (*n* = 132)

	Crude model	Demographic + health factor model	Demographic + health + social factor model
	cIRR (95% CI)	aIRR (95% CI)	aIRR (95% CI)
**Waitlist mortality rate**			
None	reference	reference	reference
Validated tool	**0.82 (0.77, 0.88)**	**0.87 (0.82, 0.94)**	**0.89 (0.83, 0.96)**
Any other tool	**0.89 (0.82, 0.97)**	0.93 (0.85, 1.01)	0.92 (0.84, 1.00)
**Transplantation rate**			
None	reference	reference	reference
Validated tool	**0.96 (0.93, 0.98)**	**0.90 (0.88, 0.93)**	**0.90 (0.88, 0.92)**
Any other tool	**0.95 (0.92, 0.98)**	**0.89 (0.86, 0.92)**	**0.90 (0.87, 0.93)**

Crude and adjusted incidence rate ratios (cIRR and aIRR) with 95% confidence intervals (CI) are presented from Poisson regression models. Demographic + health factor models adjusted for center-mean demographic (% older, % female, % Black, % Hispanic) and health factors (% with diabetes, % undergoing dialysis); demographic + health + social factor models additionally adjusted for center-mean social factors (% low education, % working for income). Associations that are statistically significant at *p* < 0.05 are bolded

### Center Characteristics of Recipients at KT

During 2017–2019, the 132 responding centers performed 76.2% of the total adult KTs in the United States. At these centers, the mean volume of adult KTs was 119.3 (SD = 81.5) per year during 2017–2019 and the center-mean age at KT was 52.6 years (SD = 2.2). KT recipients at these centers were on average 21.3% older, 38.6% female, 16.1% Hispanic, 26.5% Black, 44.4% having high school or less education, 33.1% working for income; 34.4% had diabetes, 80.5% underwent pre-KT dialysis, and 28.8% were living donation recipients. Center characteristics did not differ by frequency of frailty assessment at KT (*p* > 0.1), except that centers that always assessed frailty at KT had a lower proportion of employed recipients (*p* = 0.03) (Table 4). There were no differences in the median O/E ratios of 1-year graft loss (never = 0.99, sometimes = 1.01, always = 0.93, *p* = 0.85) and 1-year mortality (never = 0.97, sometimes = 0.93, always = 0.96, *p* = 0.53) by frailty assessment frequency (Table 4 and Supplementary Fig. S2). The proportion of centers having geriatrics program was also similar by frailty assessment frequency at KT (never = 64.4%, sometimes = 57.1%, always = 80.0%, *p* = 0.46) (Table 4).

**Table 4:** Characteristics of kidney transplant centers by frequency of frailty assessment at kidney transplantation (*N* = 132)

Center characteristics	Frequency of frailty assessment administration			
	Never (***N*** = 101)	Sometimes (***N*** = 21)	Always (***N*** = 10)	***P***-value
Adult KT volume per year	118.2 (80.4)	140.4 (88.5)	85.9 (71.1)	0.21
Age (years)	52.6 (2.2)	52.5 (1.9)	52.6 (3.5)	0.99
% Older (≥65 years)	21.2 (5.5)	21.3 (4.7)	22.0 (8.3)	0.90
% Female	38.6 (7.0)	38.9 (3.1)	37.1 (12.0)	0.79
% Hispanic	16.3 (18.4)	11.1 (11.5)	24.7 (30.2)	0.16
% Black	26.7 (18.4)	22.9 (15.2)	32.1 (25.9)	0.43
% High school or less	44.4 (10.1)	42.9 (9.8)	47.4 (13.5)	0.54
% Working for income	33.0 (11.9)	37.2 (7.8)	25.3 (13.7)	0.03
% Diabetes	34.7 (7.3)	32.4 (5.0)	35.5 (7.4)	0.34
% Undergoing dialysis	80.3 (8.1)	79.8 (8.2)	84.8 (8.1)	0.23
% Living donor transplant	29.0 (12.2)	31.4 (13.5)	21.4 (8.9)	0.11
O/E ratio of 1-year graft loss rate, median (IQR)	0.99 (0.81, 1.17)	1.01 (0.71, 1.16)	0.93 (0.77, 1.27)	0.85
O/E ratio of 1-year mortality rate, median (IQR)	0.97 (0.73, 1.29)	0.93 (0.77, 1.03)	0.96 (0.87, 1.39)	0.53
Geriatrics program, n (%)	65 (64.4%)	12 (57.1%)	8 (80.0%)	0.46
Use of a validated frailty assessment tool, n (%)	–	14 (66.7%)	8 (80.0%)	0.44

Center characteristics were gleaned from SRTR data on adult KT recipients in 2017–2019. The table shows the mean center characteristics, including characteristics that are percentages. Values are presented as mean (SD) across transplant centers unless otherwise indicated. *Abbreviations*: *KT* Kidney transplantation, *SD* Standard deviation, *IQR* Interquartile range, *SRTR* Scientific Registry of Transplant Recipients

### Frailty Assessment at KT and Post-KT Outcomes

**Fig. 2:**
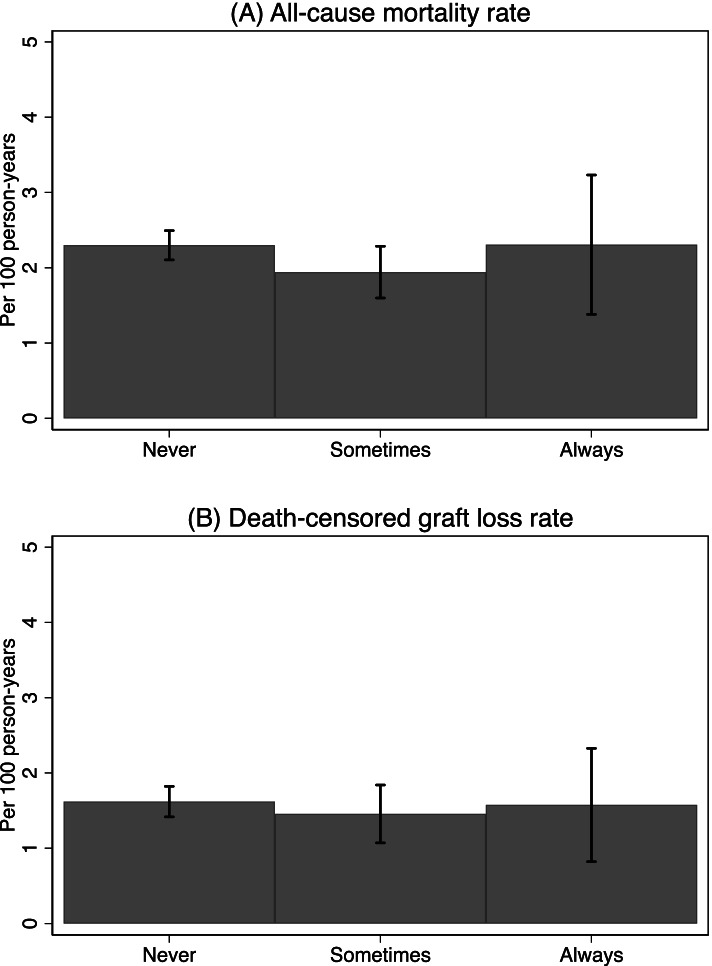
Unadjusted center-specific incidence rates among kidney transplant recipients by frequency of frailty assessment (*N* = 132)**.** Patient survival data was gleaned from SRTR data on adult KT candidates in 2017–2019. Error bars represent the corresponding 95% confidence intervals

Of the 132 centers, 7.6% (*n* = 10) always, 15.9% (*n* = 21) sometimes, and 76.5% (*n* = 101) never assessed frailty at admission for KT. Among centers assessing frailty at KT, 71.0% (*n* = 22) used a validated frailty assessment tool. The average of center median follow-up time for patient survival was 2.0 years (SD = 0.2) post-KT, with no difference by frailty assessment frequency (*p* = 0.63). These centers had a mean all-cause mortality rate of 2.2 deaths per 100 person-years (SD = 1.0); centers assessing frailty at a different frequency had similar all-cause mortality rates (*p* = 0.31) (Fig. 2A). Similarly, the average of center median follow-up time for graft survival was 1.9 years (SD = 0.2) post-KT, with no difference by frailty assessment frequency (*p* = 0.61). On average, centers had a death-censored graft loss rate of 1.6 graft losses per 100 person-years (SD = 1.0), and no difference was observed by frequency of frailty assessment at admission for KT (*p* = 0.79) (Fig. 2B).

After the adjustment for demographic, health, and social factors, centers that always (aIRR = 0.93, 95% CI: 0.76–1.13) or sometimes (aIRR = 0.93, 95% CI: 0.82–1.04) assessed frailty at KT did not differ from centers never assessing frailty in terms of post-KT patient survival rate. Yet, always assessing frailty at KT was associated with a 29% lower death-censored graft loss rate (aIRR = 0.71, 95% CI: 0.54–0.92) compared to no assessment. Centers sometimes assessing frailty had similar death-censored graft loss rates to those never assessing frailty (aIRR = 0.98, 95% CI: 0.86–1.13) (Table 5).

**Table 5:** Frailty assessment at kidney transplantation and center-specific all-cause mortality and death-censored graft loss rates (*N* = 132)

	Crude model	Demographic + health factor model	Demographic + health + social factor model
	cIRR (95% CI)	aIRR (95% CI)	aIRR (95% CI)
**All-cause mortality rate**			
Never	reference	reference	reference
Sometimes	0.89 (0.80, 1.00)	0.92 (0.82, 1.04)	0.93 (0.82, 1.04)
Always	1.01 (0.83, 1.22)	0.94 (0.78, 1.15)	0.93 (0.76, 1.13)
**Death-censored graft loss rate**			
Never	reference	reference	reference
Sometimes	0.95 (0.83, 1.08)	0.98 (0.86, 1.13)	0.98 (0.86, 1.13)
Always	**0.74 (0.57, 0.96)**	**0.71 (0.54, 0.92)**	**0.71 (0.54, 0.92)**

Crude and adjusted incidence rate ratios (cIRR and aIRR) with 95% confidence intervals (CI) are presented from Poisson regression models. Demographic + health factor models adjusted for center-mean demographic (% older, % female, % Black, % Hispanic) and health factors (% with diabetes, % undergoing dialysis, % living donor transplant); demographic + health + social factor models additionally adjusted for center-mean social factors (% low education, % working for income). Associations that are statistically significant at *p* < 0.05 are bolded

### Subgroup Analysis among Older Candidates and Recipients

Among older candidates, there was a strong association between frequency of frailty assessment and waitlist mortality rate, with a 17% lower waitlist mortality rate in centers sometimes assessing frailty at evaluation (aIRR = 0.83, 95% CI: 0.73–0.94) and a 14% lower waitlist mortality rate in centers always assessing frailty (aIRR = 0.86, 95% CI: 0.74–0.99). Compared to centers never assessing frailty at evaluation, transplantation rates in older patients were 8% lower in centers sometimes (IRR = 0.92, 95% CI: 0.87–0.98) and 18% lower in centers always (IRR = 0.82, 95% CI: 0.77–0.88) assessing frailty, respectively (Supplementary Table S1). The associations of waitlist outcomes with type of frailty assessment tool used at evaluation were strengthened among older patients: using validated frailty assessment tool was associated with 18% lower waitlist mortality rate (IRR = 0.82, 95% CI: 0.72–0.93) and 11% lower KT rate (IRR = 0.89, 95% CI: 0.84–0.95), while using any other tool was associated with 15% lower KT rate (IRR = 0.85, 95% CI: 0.79–0.92) but had no association with waitlist mortality (IRR = 0.89, 95% CI: 0.76–1.04) (Supplementary Table S2). Among older recipients, the associations of frailty assessment at admission for KT with post-KT outcomes were similar to the patterns observed among older candidates but not significant (Supplementary Table S3).

## Discussion

In this study of 132 transplant centers, representing 75.1% of all adult KT candidates and 76.2% of all adult KTs performed in the United States, we found that frailty assessments in routine clinical practices impacted pre- and post-KT outcomes. Specifically, centers always and sometimes assessing frailty at evaluation had a 9% and 11% lower waitlist mortality rate while a 6% and 12% lower transplantation rate, respectively. Yet, only using validated frailty assessment tool at evaluation was associated with lower waitlist mortality rate. Furthermore, the associations between frailty assessment practice at evaluation and waitlist outcomes were robust and had greater magnitudes among older candidates. Among recipients, centers always assessing frailty at KT had a 29% lower rate of death-censored graft loss, yet no difference in all-cause mortality rates was observed by frequency of frailty assessment at KT. However, there were no associations between frailty assessment frequency at KT and post-KT outcomes in older recipients.

Our results in KT candidates suggest that transplant centers always or sometimes assessing frailty at KT candidacy evaluation as part of clinical practice are likely to have better waitlist survival but lower transplantation rates, particularly for older candidates. This finding is consistent with prior findings from the national survey. It was reported that when a patient was identified as being frail, KT centers tend to determine the amount of social or home support before listing and prescribe prehabilitation for frail patients [6]. Thus, the better waitlist survival rate at these centers might be achieved by delivering effective interventions, such as prehabilitation [31], to frail candidates on the waitlist, by prioritizing robust older candidates, and by identifying candidates who likely improve their physiologic reserve after KT. Additionally, given the high frailty burden in KT candidates, especially in older candidates [4, 32], and the lower likelihood of receiving a KT among frail candidates [11], the lower transplantation rate at centers assessing frailty might partially result from the limited access to KT among frail candidates. However, the underlying mechanisms for this disparity are unclear.

Importantly, we found that the better waitlist survival was only associated with the use of validated frailty assessment tool; centers using any other frailty assessment tool at evaluation not only showed similar waitlist survival rates as centers not assessing frailty at all, but also had significantly lower transplantation rates, in particular among older candidates. This finding underscored that despite the demonstrated usefulness of frailty assessment in risk prediction for KT patients, the type of assessment instrument matters. The high heterogeneity of instruments to measure frailty in ESKD and KT populations has been demonstrated in several systematic reviews, and the prevalence of frailty measured by different instruments varied dramatically, ranging from 14% to 73% [33, 34]. Apart from the difference in the ability to discriminate frail patients of these assessment tools, different tools were developed on the basis of distinct frailty frameworks and are likely capturing distinct aspects of frailty. Therefore, transplant centers using frailty assessment tools that are not validated in the KT populations might not identify the most vulnerable patients and thus missed the opportunity for allocating necessary health care resources for frail patients, yet these centers could still limit the access to KT among patients being identified as “frail” by a non-validated tool. In this case, the most vulnerable older patients, suffering from both chronological aging and potentially accelerated physiological aging, are more likely to be affected. It is vital to carefully consider the ethical, financial, and medical implications of offering KT to the older population because the upper limit of age and extent of functional impairment at which the risk of transplantation outweigh the benefits are still unknown [35]. There has been an emerging need for a multidimensional approach to select ideal candidates despite age; yet routine frailty assessment based on validated instruments could potentially serve as a useful tool assisting in the process, though a standardized assessment tool tailored for KT evaluation is needed.

In KT recipients, we found a lower rate of graft loss, but not mortality, among centers always assessing frailty at admission for KT. Frailty assessments at KT admission could make healthcare providers aware of which patients need a tailored, patient-centered care for prevention of adverse transplant outcomes. Moreover, the lower graft loss rate might be a combined result from practices pre- and post-KT, as all the 10 centers always assessing frailty at KT also reported assessing frailty at candidacy evaluation (3 sometimes and 7 always). There has been evidence showing that frailty status, as measured by the PFP, is dynamic between evaluation and KT and transitions to more frail states are associated with poor post-KT outcomes [36]. By assessing frailty at both critical time points, these centers had better opportunities to identify patients who most likely benefit from pre-KT prehabilitation [31] and post-KT rehabilitation [37].

Our findings arise from the practices and outcomes of transplant centers that impact 75% of KT candidates and 76% of recipients in the United States. To our knowledge, this is the first study to date exploring the association of frailty assessments in clinical practices with center-level pre- and post-KT outcomes. There are a number of notable strengths of this study, including the novel analytic approach to explore the center-level associations, the ability to identify type of frailty assessment tool and to further study the association with use of a validated tool, separate analyses among older patients, geographic diversity of the responding centers, and the high percentages of KT candidates and recipients reflected by the results of this study.

This study is limited by the transplant centers included. For example, we were unable to examine the outcomes of centers that assessed frailty solely at KT admission, since no such centers were included in the survey. However, it is likely that few transplant centers assess frailty only at KT, since only 61% of centers agreed that frailty assessments should be used in decisions for the timing of KT, while 96% of centers agreed on the use in decisions regarding candidate selection [6]. Another limitation is the cross-sectional design; our findings do not support causal inferences between frailty assessment practices and clinical outcomes of transplant centers. In addition, although we accounted for all available center-level characteristics and tested the center performances (O/E ratios) and the presence of a geriatrics program at each center, there may be residual confounding effects. This may result from unmeasurable factors, such as how frailty assessment results were used in transplant care, the extent to which a negative result may affect clinical decision-making for the patient, and what additional healthcare services will be triggered by negative results. However, our results suggested that better quality-controlled centers are not the only centers that assess frailty for transplant decision-making. Furthermore, there were a wide variety of tools to measure frailty for KT patients in clinical practice, and the most commonly used tools were a timed walk test and measuring body mass index [6]. Although we examined the effects of using a validated frailty assessment tool at evaluation, the sample size did not allow the analysis of using a validated tool at KT, nor further subgroup analysis of specific frailty assessment tools or further adjustment for type of tools in the model of frailty assessment frequency. Thus, future studies should identify which measure is best suited for KT patients.

## Conclusions

In summary, our findings suggest that, regardless of center characteristics, assessing frailty as part of KT candidacy evaluation is associated with a lower transplantation rate, but using a validated frailty assessment tool is also associated with better waitlist survival, in particular among older candidates. Centers always assessing frailty at admission for KT are likely to have a better graft survival, while it is unclear whether the lower graft loss rate resulted from frailty assessments at a single time point at admission or at both evaluation and admission for KT. This study presents the potential usefulness of using validated frailty assessments in clinical practice for KT patients. Based on our findings, we recommend that transplant centers may utilize frailty assessment instruments that have been validated in KT populations as a tool to secure KT access for appropriate candidates despite age and to better allocate health care resources for patients identified as frail, particularly for older patients. For example, the transplant team may adapt the well-developed geriatric care frameworks to the setting of KT care, developing conversations with the family members of KT patients to achieve a more favorable transplant outcome. Also, healthcare providers in other clinical specialties should be aware of the potential utility of frailty assessments having been validated in the target populations, even among younger patients. Further research should explore the optimal subpopulations and procedures for assessing frailty in clinical practice.

## Supplementary Information

**Additional file 1.**

## Data Availability

The datasets used and/or analyzed during the current study are available from the corresponding author on reasonable request.

## Abbreviations

95% CI: 95% confidence interval
aIRR: Adjusted incidence rate ratio
ANOVA: Analysis of variance
AST: American Society of Transplantation
cIRR: Crude incidence rate ratio
ESKD: End-stage kidney disease
IRR: Incidence rate ratio.
KT: Kidney transplant
O/E ratio: Observed to expected ratio
OPTN: Organ Procurement and Transplantation Network
PFP: Physical Frailty Phenotype
SD: Standard deviation
SPPB: Short Physical Performance Battery
SRTR: Scientific Registry of Transplant Recipients
