# Supplementary material for: Transplant centers that assess frailty as part of clinical practice have better outcomes
Source: BMC Geriatr. 2022 Jan 27;22:82. doi: 10.1186/s12877-022-02777-2 (PMC8793239; doi:10.1186/s12877-022-02777-2)
Supplement: Supplementary file 1 — Additional file 1. [file 12877_2022_2777_MOESM1_ESM.docx]

**SUPPLEMENTAL MATERIALS FOR**

**Transplant Centers That Assess Frailty as Part of Clinical Practice Have Better Outcomes**

**Authors:**

Xiaomeng Chen MSPH (1), Yi Liu ScM (1), Valerie Thompson BA (1), Nadia M. Chu PhD MPH (1,2), Elizabeth King MD PhD (1), Jeremy D. Walston MD (3), Jon A. Kobashigawa MD (4), Darshana M. Dadhania MD (5), Dorry L. Segev MD PhD (1,2), Mara A. McAdams-DeMarco PhD (1,2)

1. Department of Surgery, Johns Hopkins University School of Medicine, Baltimore, MD, USA.
2. Department of Epidemiology, Johns Hopkins Bloomberg School of Public Health, Baltimore, MD, USA.
3. Department of Medicine, Johns Hopkins School of Medicine, Baltimore, MD, USA.
4. Comprehensive Transplant Center, Cedars-Sinai Medical Center, Los Angeles, CA, USA.
5. Division of Nephrology and Hypertension, Weill Cornell Medicine, New York, NY, USA.

**Table of Contents:**

**Supplementary Figure S1:** Distributions of center-specific observed to expected (O/E) ratios for adverse outcomes by frequency of frailty assessment at kidney transplant candidacy evaluation (N=132).

**Supplementary Figure S2:** Distributions of center-specific observed to expected (O/E) ratios for adverse outcome rates by frequency of frailty assessment at kidney transplantation (N=132).

**Supplementary Table S1:** Frailty assessment at kidney transplant candidacy evaluation and center-specific waitlist mortality and transplantation rates in older candidates (n=132)

**Supplementary Table S2:** Type of frailty assessment tool at kidney transplant evaluation and center-specific waitlist mortality and transplantation rates in older recipients (n=132)

**Supplementary Table S3:** Frailty assessment at kidney transplantation and center-specific all-cause mortality and death-censored graft loss rates in older recipients (n=132)

**Supplementary Figure S1:** Distributions of center-specific observed to expected (O/E) ratios for adverse outcomes by frequency of frailty assessment at kidney transplant candidacy evaluation (N=132).

The center-specific O/E ratios were gleaned from the biannual SRTR Program-Specific Report (PSR) released in 10/2018. The reference population of the estimates was all KT patients on the waiting list at any time during 12/31/2015 – 12/30/2017.

**(A) Waitlist mortality rate**


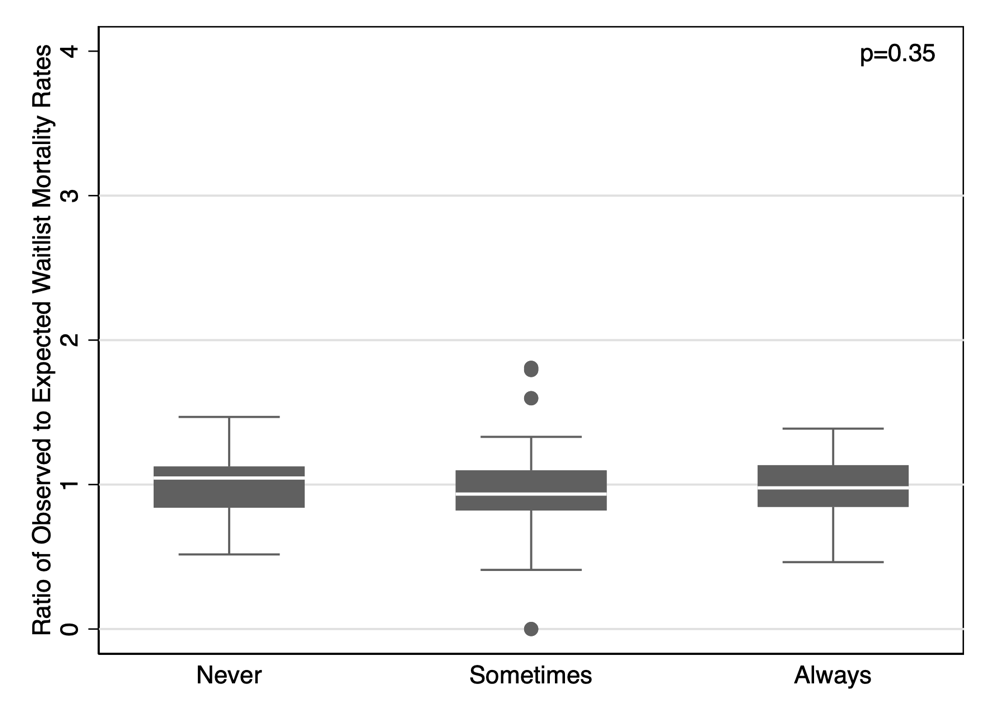


**(B) Transplantation rate**


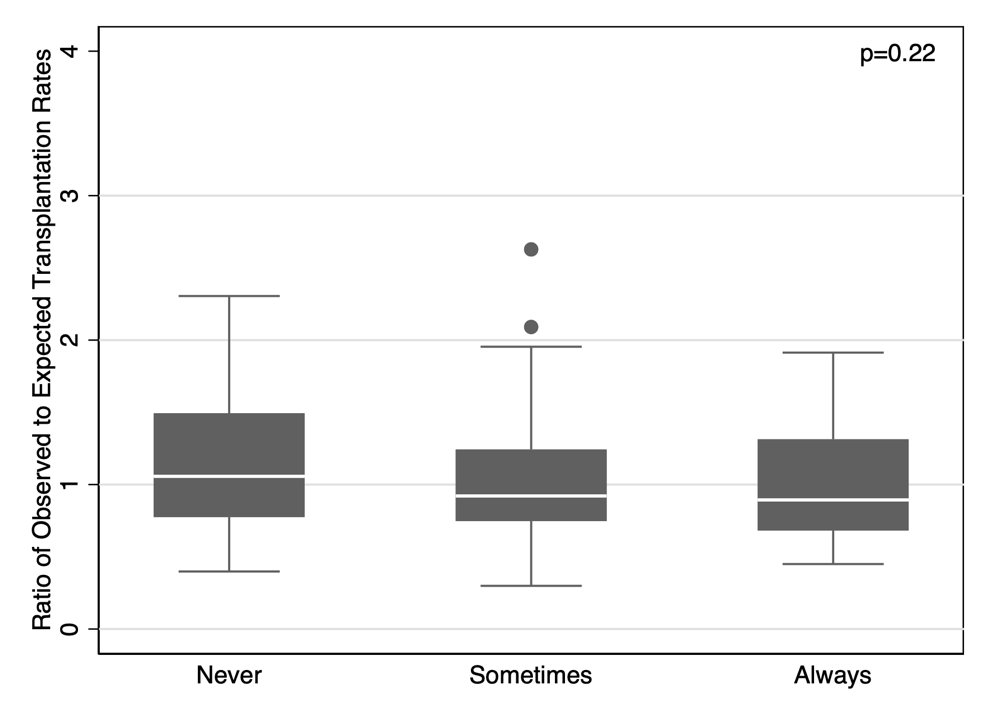


**Supplementary Figure S2:** Distributions of center-specific observed to expected (O/E) ratios for adverse outcome rates by frequency of frailty assessment at kidney transplantation (N=132).

The center-specific O/E ratios were gleaned from the biannual SRTR Program-Specific Report (PSR) released in 10/2018. The reference population of the estimates was all KT patients undergoing transplant during 12/31/2015 – 12/30/2017.

**(A) 1-year all-cause mortality rate**


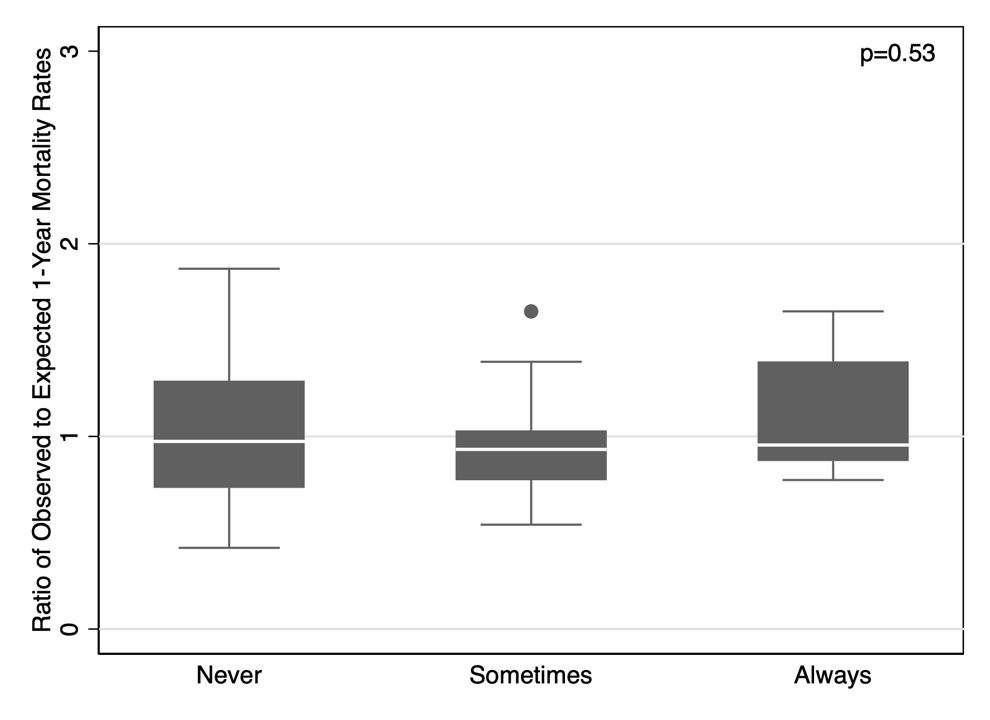


**(B) 1-year graft loss rate (including death)**


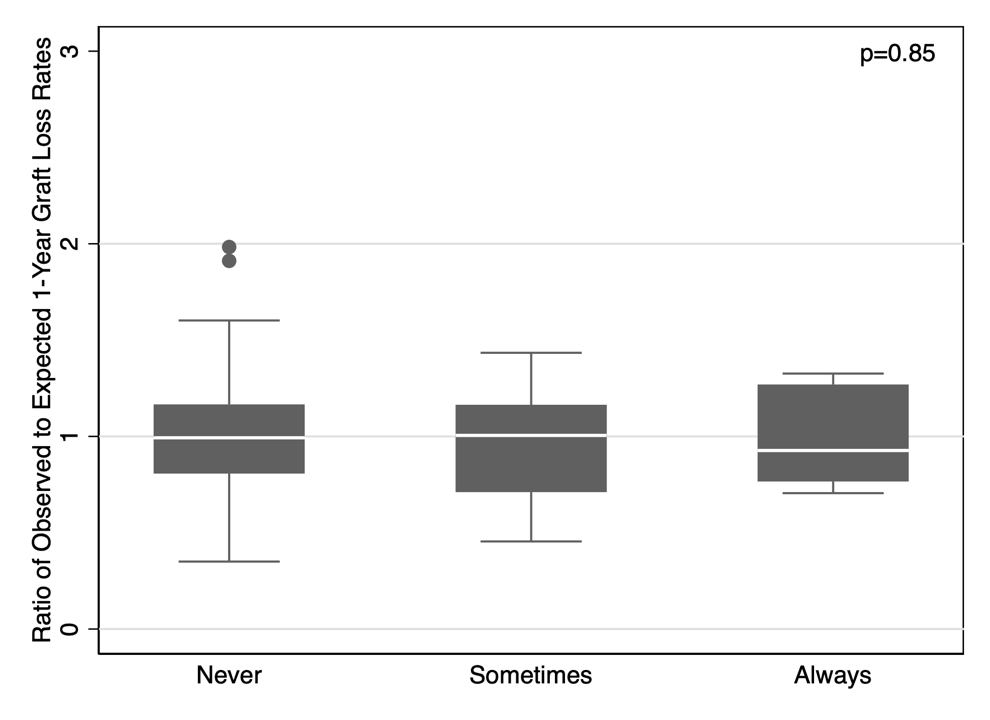


**Supplementary Table S1:** Frailty assessment at kidney transplant candidacy evaluation and center-specific waitlist mortality and transplantation rates in older candidates (N=132)

|  | **Crude model** | **Demographic + health factor model** | **Demographic + health + social factor model** |
| --- | --- | --- | --- |
|  | cIRR (95% CI) | aIRR (95% CI) | aIRR (95% CI) |
| **Waitlist mortality rate** | | | |
| Never | reference | reference | reference |
| Sometimes | **0.82 (0.72, 0.93)** | **0.82 (0.72, 0.94)** | **0.83 (0.73, 0.94)** |
| Always | **0.83 (0.72, 0.95)** | **0.86 (0.74, 0.99)** | **0.86 (0.74, 0.99)** |
| **Transplantation rate** | |  |  |
| Never | reference | reference | reference |
| Sometimes | **0.93 (0.88, 0.99)** | **0.91 (0.86, 0.96)** | **0.92 (0.87, 0.98)** |
| Always | **0.88 (0.82, 0.94)** | **0.83 (0.77, 0.88)** | **0.82 (0.77, 0.88)** |

Crude and adjusted incidence rate ratios (cIRR and aIRR) with 95% confidence intervals (CI) are presented from Poisson regression models. Demographic + health factor models adjusted for center-mean demographic (% female, % Black, % Hispanic) and health factors (% with diabetes, % undergoing dialysis); demographic + health + social factor models additionally adjusted for center-mean social factors (% low education, % working for income). Associations that are statistically significant at p<0.05 are bolded.

**Supplementary Table S2:** Type of frailty assessment tool at kidney transplant evaluation and center-specific waitlist mortality and transplantation rates in older recipients (n=132)

|  | **Crude model** | **Demographic + health factor model** | **Demographic + health + social factor model** |
| --- | --- | --- | --- |
|  | cIRR (95% CI) | aIRR (95% CI) | aIRR (95% CI) |
| **Waitlist mortality rate** | | | |
| None | reference | reference | reference |
| Validated tool | **0.80 (0.71, 0.90)** | **0.81 (0.72, 0.92)** | **0.82 (0.72, 0.93)** |
| Any other tool | 0.87 (0.75, 1.02) | 0.89 (0.76, 1.04) | 0.89 (0.76, 1.04) |
| **Transplantation rate** | |  |  |
| None | reference | reference | reference |
| Validated tool | **0.92 (0.87, 0.97)** | **0.88 (0.83, 0.93)** | **0.89 (0.84, 0.95)** |
| Any other tool | **0.90 (0.84, 0.97)** | **0.86 (0.80, 0.92)** | **0.85 (0.79, 0.92)** |

Crude and adjusted incidence rate ratios (cIRR and aIRR) with 95% confidence intervals (CI) are presented from Poisson regression models. Demographic + health factor models adjusted for center-mean demographic (% female, % Black, % Hispanic) and health factors (% with diabetes, % undergoing dialysis); demographic + health + social factor models additionally adjusted for center-mean social factors (% low education, % working for income). Associations that are statistically significant at p<0.05 are bolded.

**Supplementary Table S3:** Frailty assessment at kidney transplantation and center-specific all-cause mortality and death-censored graft loss rates in older recipients (N=132)

|  | **Crude model** | **Demographic + health factor model** | **Demographic + health + social factor model** |
| --- | --- | --- | --- |
|  | cIRR (95% CI) | aIRR (95% CI) | aIRR (95% CI) |
| **All-cause mortality rate** | | | |
| Never | reference | reference | reference |
| Sometimes | 1.02 (0.86, 1.22) | 1.04 (0.87, 1.25) | 1.05 (0.87, 1.26) |
| Always | 0.97 (0.69, 1.36) | 0.92 (0.65, 1.30) | 0.89 (0.63, 1.26) |
| **Death-censored graft loss rate** | | | |
| Never | reference | reference | reference |
| Sometimes | 0.84 (0.63, 1.12) | 0.85 (0.63, 1.14) | 0.86 (0.64, 1.15) |
| Always | 0.66 (0.36, 1.21) | 0.61 (0.33, 1.13) | 0.60 (0.32, 1.12) |

Crude and adjusted incidence rate ratios (cIRR and aIRR) with 95% confidence intervals (CI) are presented from Poisson regression models. Demographic + health factor models adjusted for center-mean demographic (% female, % Black, % Hispanic) and health factors (% with diabetes, % undergoing dialysis, % living donor transplant); demographic + health + social factor models additionally adjusted for center-mean social factors (% low education, % working for income). Associations that are statistically significant at p<0.05 are bolded.
